# Grape-Seed Proanthocyanidin Extract Reverts Obesity-Related Metabolic Derangements in Aged Female Rats

**DOI:** 10.3390/nu13062059

**Published:** 2021-06-16

**Authors:** Marta Sierra-Cruz, Alba Miguéns-Gómez, Carme Grau-Bové, Esther Rodríguez-Gallego, Mayte Blay, Montserrat Pinent, Anna Ardévol, Ximena Terra, Raúl Beltrán-Debón

**Affiliations:** MoBioFood Research Group, Department of Biochemistry and Biotechnology, Universitat Rovira i Virgili, 43007 Tarragona, Spain; marta.sierra@urv.cat (M.S.-C.); alba.miguens@urv.cat (A.M.-G.); carme.grau@urv.cat (C.G.-B.); esther.rodriguez@urv.cat (E.R.-G.); mteresa.blay@urv.cat (M.B.); montserrat.pinent@urv.cat (M.P.); anna.ardevol@urv.cat (A.A.); raul.beltran@urv.cat (R.B.-D.)

**Keywords:** obesity, ageing, metabolic syndrome, proanthocyanidins, adiposity, liver steatosis

## Abstract

Obesity and ageing are current issues of global concern. Adaptive homeostasis is compromised in the elderly, who are more likely to suffer age-related health issues, such as obesity, metabolic syndrome, and cardiovascular disease. The current worldwide prevalence of obesity and higher life expectancy call for new strategies for treating metabolic disorders. Grape-seed proanthocyanidin extract (GSPE) is reported to be effective in ameliorating these pathologies, especially in young animal models. In this study, we aimed to test the effectiveness of GSPE in modulating obesity-related pathologies in aged rats fed an obesogenic diet. To do so, 21-month-old rats were fed a high-fat/high-sucrose diet (cafeteria diet) for 11 weeks. Two time points for GSPE administration (500 mg/kg body weight), i.e., a 10-day preventive GSPE treatment prior to cafeteria diet intervention and a simultaneous GSPE treatment with the cafeteria diet, were assayed. Body weight, metabolic parameters, liver steatosis, and systemic inflammation were analysed. GSPE administered simultaneously with the cafeteria diet was effective in reducing body weight, total adiposity, and liver steatosis. However, the preventive treatment was effective in reducing only mesenteric adiposity in these obese, aged rats. Our results confirm that the simultaneous administration of GSPE improves metabolic disruptions caused by the cafeteria diet also in aged rats.

## 1. Introduction

The prevalence of obesity has been increasing worldwide over the last 30 years [1]. Obesity is associated with low-grade inflammation and metabolic syndrome, which is characterized by alterations in glucose, fatty acids, and amino acid metabolism and leads both to a decrease in insulin sensitivity and a decline in one’s ability to adjust to energy availability [2].

The prevalence of obesity is increasing steadily among the aged population [3] at a time when the number and proportion of older people are growing worldwide. By 2050, there will be roughly two billion people over the age of 60 [4]. Ageing is associated with the progressive loss of physiological functions [5] as well as metabolic alterations, such as increases in (1) abdominal white adipose tissue, (2) fat deposition in skeletal muscle and the liver, and (3) the expression of pro-inflammatory cytokines, all of which lead to a decrease in insulin sensitivity [5]. Together, obesity and ageing contribute to the development of associated diseases, mainly type-2 diabetes, cardiovascular diseases, and several types of cancer.

Since obesity contributes directly to the ageing process [6], effective anti-obesity treatments are needed to improve the quality of life of the elderly population. Traditional strategies have been based on physical exercise and dietary interventions. Studies on caloric restriction conducted in animal models have shown that this type of intervention increases life expectancy [7]. More specifically, caloric restriction can extend the life expectancy of mice and rats by 50% compared to control animals fed ad libitum [8,9]. However, weight loss in old, obese adults can also lead to a high loss of skeletal muscle or bone mass, which can be detrimental [4]. There is, therefore, an urgent need for innovation in therapeutic interventions aimed at treating obesity and ageing-related processes, mainly to improve quality of life and increase life expectancy. One interesting strategy that targets the elderly obese in this context is based on food bioactive compounds.

Food bioactive compounds are components (found in small quantities) of plants and lipid-rich foods [10]. An important example of these compounds are polyphenols. A group of polyphenols with potential beneficial effects on human health are proanthocyanidins (PACs) [11]. These are oligomers and polymers of monomeric flavan-3-ols mainly found in everyday food and beverages such as grapes, cocoa, chocolate, red wine, and green tea [11,12,13]. The literature shows controversial results regarding the effect of PACs on adiposity and body weight. While some studies have shown that PACs may lead to a decrease in body weight by up-regulating energy expenditure-related genes, others have reported no such effectiveness [12,14,15]. Standardized methods, doses, and times of administration are therefore required. Apart from body weight modulation, several beneficial effects of PACs have been demonstrated in young experimental animals, including (1) a decrease in fatty acid synthesis and fat uptake and an increase in energy expenditure in skeletal muscle and the liver [13,15]; (2) a modulation of the neuropeptides involved in food intake and satiety [13]; (3) an inhibition of digestive enzymes, mainly amylase and lipase, which leads to a reduction in lipid and glucose absorption from the gut [12,13]; (4) an antioxidant beneficial effect on inflammatory processes through reduction in the activity of antioxidant enzymes, such as catalase or superoxide dismutase (SOD) [12]; and (5) hypolipidemic and hypotriglyceridemic effects that lead to an improvement in lipid metabolism and the attenuation of hepatic steatosis in mice fed a high-fat diet [16] and rabbits fed a high-fat, high-cholesterol diet [17].

These studies have then demonstrated that grape-seed proanthocyanidin extract (GSPE) shows potential beneficial effects against obesity and metabolic-related pathologies in young and adult animal models [18,19,20]. However, little is known about the potential benefits of GSPE in improving metabolic alterations in the elderly. Therefore, with this background and given the relevance of the increasing prevalence of the aged-obese population, it becomes essential to take actions to promote healthy ageing where PACs might have a role. We have previously evaluated the effects of a pharmacological dose of GSPE on young rats under an obesogenic challenge and found that GSPE reverted several features of the metabolic syndrome [21]. As far as we know, this is the first study to evaluate the effects of a PAC extract against both obesity and ageing. In this study we analyse the potential beneficial effects of an oral administration of a grape-seed proanthocyanidin extract on body weight gain and homeostatic buffering loss linked to obesity and ageing when administered either as a preventive or simultaneous treatment to aged rats fed an obesogenic diet.

## 2. Materials and Methods

### 2.1. Proanthocyanidin Extract

The grape-seed extract rich in proanthocyanidins (GSPE) was provided by Les Dérivés Résiniques et Terpéniques (Dax, France). According to the manufacturer, the GSPE used in this study (batch number: 207100) contains a total procyanidin content of 76.9% and consists of a mixture of monomers (23.1%), dimers (21.7%), trimers (21.6%), tetramers (22.2%), and pentamers (11.4%) of flavan-3-ols.

### 2.2. Animal Model

A total of 42 aged Wistar female rats (21 months old), each weighing 300–350 g, were acquired from Charles River Laboratories (Barcelona, Spain). After one week of adaptation, the rats were individually housed in the animal quarters at 22 °C with a 12-h light/12-h dark cycle and fed a standard chow diet (Teklad 2014 Envigo, Barcelona, Spain) ad libitum and tap water. Laboratory rats start reproductive senescence at approximately 20 months old [22]. According to the age correlation with humans at this period of life, our model of study could be useful to understand alterations linked to obesity and ageing in 60-year-old humans. The rats were then randomly divided into four experimental groups (*n* = 14) and fed a standard chow diet ad libitum. The control group (STD) received only the standard chow diet throughout the experiment. In addition to the standard chow diet, the other groups received a cafeteria diet as a model of a high-fat/high-sucrose diet (CAF groups). The cafeteria diet consisted of bacon, sausages, paté and biscuits, carrots, muffins, and sugared milk, which induces voluntary hyperphagia [23]. This diet was offered freshly ad libitum every day for 75 days. The energy contents of the meals offered to the animals are shown in Table 1.

**Figure 1:**
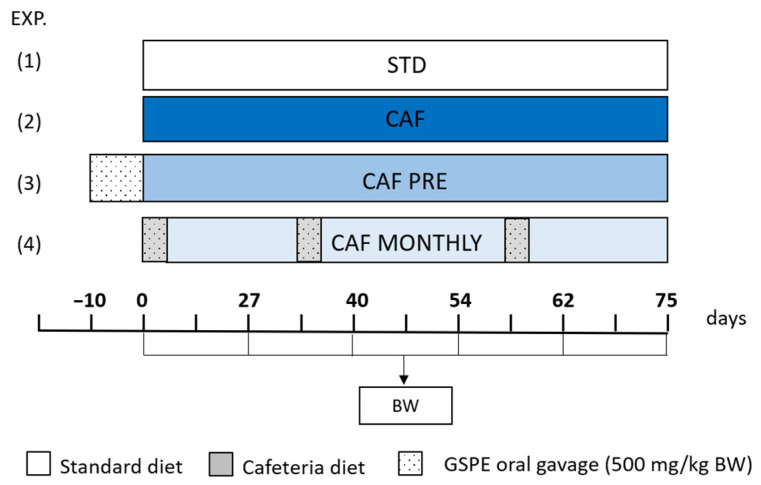
Schematic diagram of the experimental design. All groups were adapted to the environment and to oral gavage during one week before experiment started. Body weight was measured every two weeks. (1) STD: rats receiving standard diet during the whole experiment; (2) CAF: rats receiving standard diet before cafeteria diet intervention; (3) CAF PRE: rats receiving GSPE preventive treatment for 10 days before the cafeteria diet intervention started; (4) CAF MONTHLY: rats receiving a 5-day GSPE treatment simultaneously with the cafeteria diet once per month. Abbreviations: CAF, cafeteria diet; GSPE, grape seed proanthocyanidin extract; BW, Body weight.

Two of the cafeteria-fed groups were supplemented with GSPE. An oral dose of 500 mg/kg BW (body weight) was administered (1) as a preventive treatment for 10 days prior to the cafeteria diet intervention (CAF PRE) and (2) simultaneously with the cafeteria diet for 5 days once per month (CAF MONTHLY). The GSPE was dissolved in tap water and administered as an oral gavage to the animals at 6 pm, three hours after all available food had been removed. Fresh food was given to the animals one hour after they received the GSPE dose. The animals that did not receive supplementation with GSPE received water as a vehicle. The experimental design is illustrated in Figure 1.

### 2.3. Blood and Tissue Collection

At the end of the study, the animals were euthanized by decapitation after they had fasted for 12 h. The blood was collected using heparin (Deltalab, Barcelona, Spain) as an anticoagulant. Plasma was obtained by centrifugation (1500× *g*, 15 min, 4 °C) and stored at −80 °C until analysis. White adipose tissue depots (retroperitoneal (rWAT), mesenteric (mWAT), and periovaric (oWAT)), brown adipose tissue (BAT), and the liver, kidneys, and spleen were rapidly removed, weighed, snap-frozen in liquid nitrogen, and stored at −80 °C.

### 2.4. Morphometric and Biochemical Variables

Body weight was monitored twice a month. Commercial colorimetric enzyme kits were used to measure the concentrations of plasma glucose, triacylglycerol (TAG), cholesterol, urea, creatinine (QCA, Tarragona, Spain), non-esterified fatty acids (NEFAs, Wako, Neuss, Germany), and β-hydroxybutyrate (Ben Biochemical Enterprise, Milan, Italy). Commercial ELISA kits were used to quantify plasma levels of insulin (Millipore, Madrid, Spain), glucagon (Mercodia, Uppsala, Sweden), tumour necrosis factor-α (TNF-α), and interleukin-6 (IL-6) (Thermo Scientific, Spain). Homeostatic model assessment of insulin resistance (HOMA-IR) and homeostasis model assessment of β-cell dysfunction (HOMA-β) were calculated using glucose and insulin fasting values.

Liver homogenization was performed in a tissue lyser (50 s, 2 cycles at maximum potency) using a 0.1% Triton X-100 phosphate-buffered solution after 4 °C centrifugation. The supernatant collected was used for further triacylglycerol measurement.

### 2.5. Analysis of Liver Steatosis

Liver samples were added to a 4% formaldehyde solution for 24 h and transferred to a 70% ethanol solution until paraffin inclusion. Tissue sections 4 µm thick were cut from paraffin blocks and placed on glass slides. Haematoxylin and eosin (H&E) staining was performed using standard procedures. These sections were analysed under a light microscope to detect changes in tissue architecture.

Samples were scored 0–3 according to the percentage of hepatocytes affected by fatty infiltration (0: <5%; 1: 6–33%; 2: 34–66%; 3: 67–100% of the surface). The histological diagnosis of liver steatosis was based on the criteria described by Brunt et al. [24]. Scoring was done blindly by a specialist.

### 2.6. Statistical Analysis

Body weight and morphometric variables are represented as the mean ± standard error of the mean (SEM). Kruskal–Wallis and Mann–Whitney non-parametric statistical tests were assessed. Analyses were performed with XLSTAT 2020.1 (Addinsoft, Spain). *p*-values < 0.05 were considered statistically significant.

## 3. Results

### 3.1. GSPE Prevents Body Weight Increase in Obesogenic Conditions When Administered Simultaneously with the Cafeteria Diet

To evaluate the effects of GSPE in aged rats after an obesogenic challenge, such as the cafeteria diet, we first tested its effectiveness on body weight gain.

**Figure 2:**
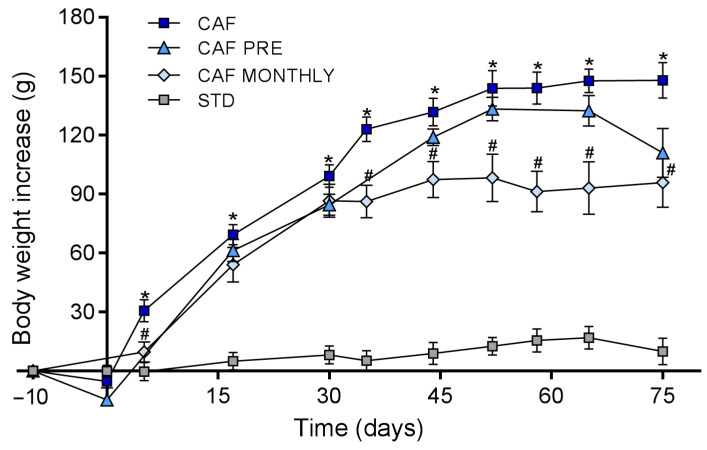
Body weight gain during the experiment. STD: lean rats fed a standard diet; CAF: rats fed a cafeteria diet; CAF PRE: rats receiving preventive treatment of GSPE during 10 days before cafeteria diet intervention; CAF MONTHLY: rats receiving GSPE treatment during 5 days once per month simultaneously fed with cafeteria diet. Values are means ± SEM. * *p*-value < 0.05 compared to STD rats. # *p*-value < 0.05 compared to CAF rats.

Figure 2 shows that ingesting a cafeteria diet significantly increased body weight gain from day 15 to the end of the experiment (day 75). CAF MONTHLY rats, which received GSPE once a month simultaneously with the cafeteria diet, showed a significant decrease in body weight gain from day 35 to the end of the experiment and reached an 8.4% greater reduction than rats in the CAF group (Figure 2). The body weight gain of CAF PRE rats was also lower than that of rats in the CAF group throughout the experiment. However, this difference did not reach statistical significance.

We have previously reported that GSPE acutely reduces food intake [25]. We measured food intake on the days that included GSPE or vehicle treatment. Cafeteria diet consumption significantly increased food intake compared to chow diet (485 vs. 198 kJ/day, *p* < 0.0001). GSPE significantly reduced food intake when administered with cafeteria diet (CAF vs. CAF MONTHLY; 485 vs. 343 kJ/day, *p* < 0.0001). In the 10-day pre-treatment with GSPE, the animals slightly reduced their food intake (CAF vs. CAF PRE groups before cafeteria diet onset; 175 vs. 141 kJ/day, *p* < 0.0001).

### 3.2. Preventive and Simultaneous GSPE Treatments Reduced Mesenteric Adiposity

The effect of GSPE treatments on several morphometric variables is described in Table 2. As expected, the obesogenic diet increased body weight and expanded white adipose tissue depots in CAF rats when compared to STD rats. Brown adipose tissue weight also increased in CAF rats compared to STD rats. Taken together, the cafeteria diet increased the adiposity index of CAF rats compared to control rats. Moreover, the obesogenic diet induced weight gain in the liver, spleen, and kidneys.

Both preventive and simultaneous GSPE treatments reduced mesenteric adipose tissue weight. Moreover, CAF MONTHLY rats experienced a significant reduction in visceral adiposity compared to CAF rats.

No differences were observed in the weight of the liver, spleen, or kidneys between rats in the GSPE-treated groups and CAF rats.

### 3.3. GSPE Effects on the Glucidic and Lipidic Profile of Obese, Aged Rats

Since obesity and ageing are linked to metabolic dysfunctions, such as dyslipidaemia and insulin resistance, we also analysed the metabolic state of the animals after they were subjected to the obesogenic challenge.

**Figure 3:**
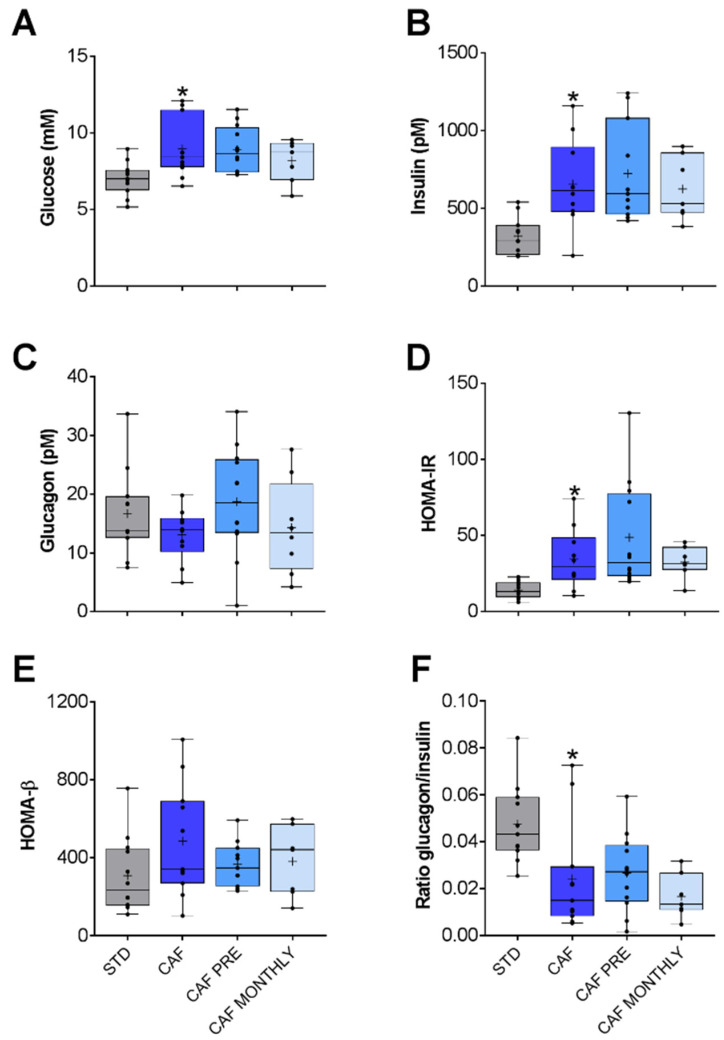
Glucose metabolism-related biochemical parameters: (**A**) Glucose. (**B**) Insulin. (**C**) Glucagon. (**D**) HOMA-IR. (**E**) HOMA-β. (**F**) Glucagon/insulin ratio. STD: lean rats fed a standard diet; CAF: rats fed a cafeteria diet; CAF PRE: rats receiving preventive treatment of GSPE during 10 days before cafeteria diet intervention; CAF MONTHLY: rats receiving GSPE treatment during 5 days once per month simultaneously fed with cafeteria diet. Values are represented as boxplots showing the median and the IQR. Means are represented as +. * *p*-value < 0.05 compared to STD rats.

Glucidic profile was altered due to the cafeteria diet. Glucose and insulin levels increased significantly in the CAF group compared to the STD group (Figure 3A,B, respectively).

To explore this profile in greater detail, we also determined the HOMA-IR and HOMA-β ratios. HOMA-IR measures insulin-resistance in Wistar rats [26] while HOMA-β measures β-cell function both on the basis of fasting glucose and insulin levels. In this study, HOMA-IR was significantly higher in the CAF group than in the STD group (Figure 3D), but no differences were observed in HOMA-β values (Figure 3E). Since insulin and glucagon hormones have opposite functions, we also calculated the glucagon/insulin ratio in order to better understand the balance between catabolism and anabolism. Although the cafeteria diet did not lead to a significant reduction in glucagon levels in plasma, the glucagon/insulin ratio was lower in the CAF group than in the control group (Figure 3F). Taken together, aged rats fed an obesogenic diet presented higher insulin resistance than aged rats fed a standard diet. However, GSPE was unable to ameliorate this condition (Figure 3).

**Figure 4:**
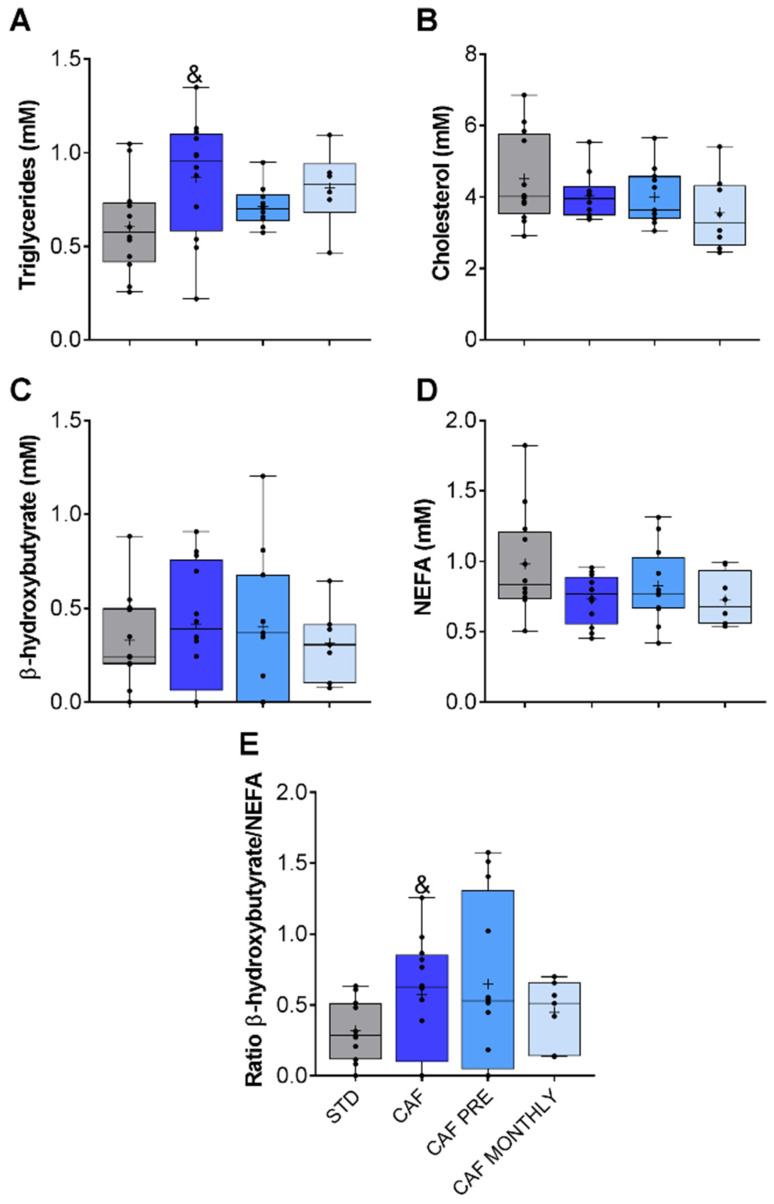
Lipid metabolism-related biochemical parameters. (**A**) Triglyceride levels. (**B**) Cholesterol levels. (**C**) β-hydroxybutyrate levels. (**D**) NEFA levels. (**E**) β-hydroxybutyrate/NEFA ratio. STD: lean rats fed a standard diet; CAF: rats fed a cafeteria diet; CAF PRE: rats receiving preventive treatment of GSPE during 10 days before cafeteria diet intervention; CAF MONTHLY: rats receiving GSPE treatment during 5 days once per month simultaneously fed with cafeteria diet; NEFA, non-esterified fatty acids). Values are represented as boxplots showing the median and the IQR. Means are represented as +. Trends: ^&^ 0.05 < *p*-value < 0.1 compared to STD rats.

With regard to lipid metabolism, CAF rats had higher TAG levels (Figure 4A), but no differences were observed in cholesterol, β-hydroxybutyrate, or NEFA parameters (Figure 4B–D, respectively). We also determined the β-hydroxybutyrate/NEFA ratio in order to evaluate the ketogenesis rate. Only the cafeteria diet tended to increase this rate (Figure 4E).

Neither preventive nor simultaneous GSPE supplementation had any effect on lipid metabolism-related parameters (Figure 4).

### 3.4. Renal Function in Aged Rats Fed the Cafeteria Diet Is Not Modified by the Obesogenic Diet or GSPE Treatments

**Figure 5:**
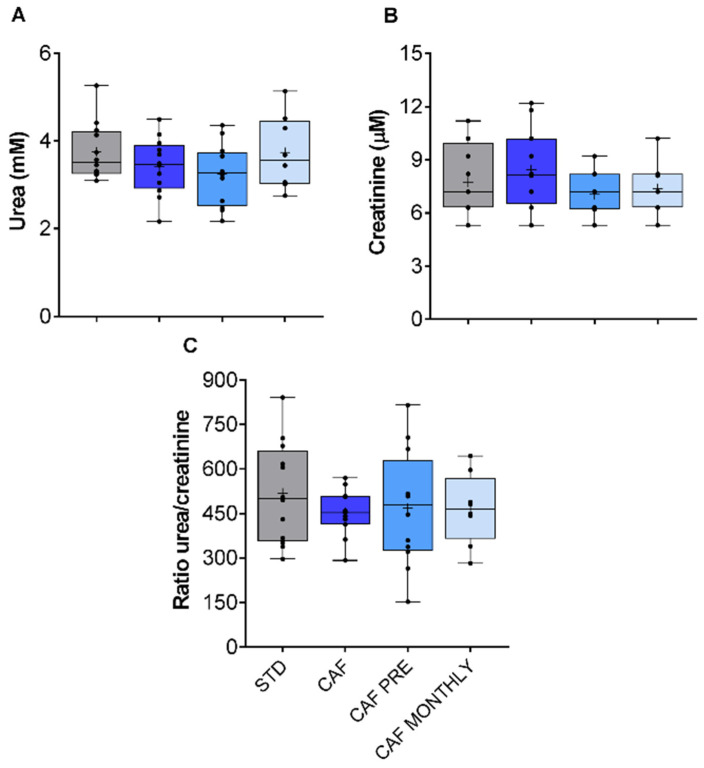
Renal metabolism-related biochemical parameters. (**A**) Urea levels. (**B**) Creatinine levels. (**C**) Urea/creatinine ratio. STD: lean rats fed a standard diet; CAF: rats fed a cafeteria diet; CAF PRE: rats receiving preventive treatment of GSPE during 10 days before cafeteria diet intervention; CAF MONTHLY: rats receiving GSPE treatment during 5 days once per month simultaneously fed with cafeteria diet. Values are represented as boxplots showing the median and the IQR. Means are represented as +.

The kidneys are one of the organs that are most sensitive to age-related changes, especially those that affect renal plasma flow (RPF) and the glomerular filtration rate (GFR) [27]. Moreover, obesity is widely reported to have biological consequences on kidney function in certain experimental animals [28]. Since neither the cafeteria diet nor GSPE supplementation affected urea or creatinine levels, no effects on kidney function were observed (Figure 5A,B, respectively).

### 3.5. GSPE Reduces Liver Lipid Content in Obese, Aged Rats

**Figure 6:**
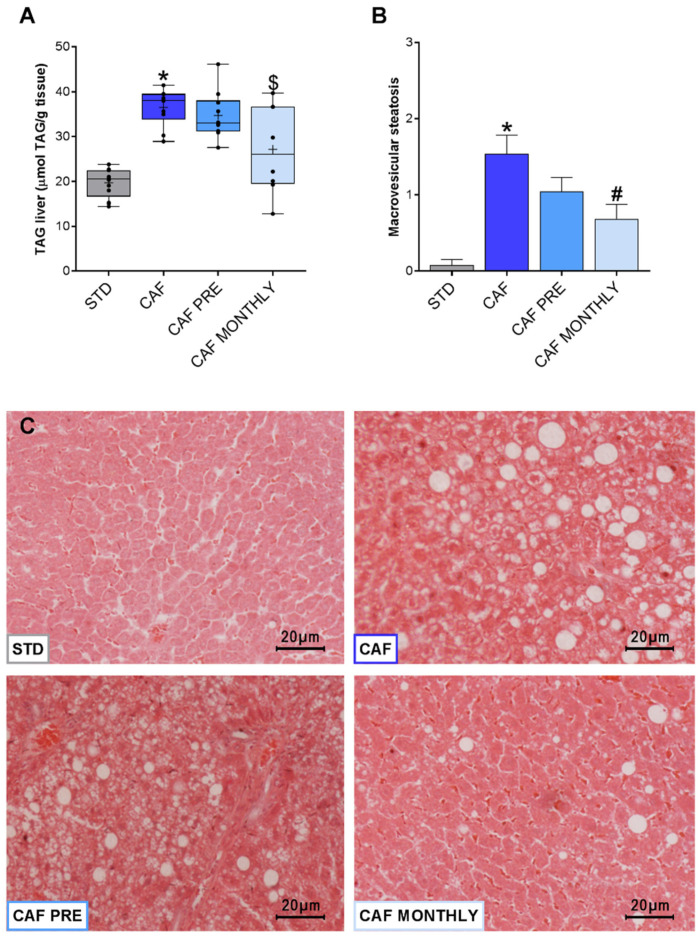
Effects of GSPE treatment on liver steatosis. (**A**) Simultaneous treatment with GSPE reduces liver lipid accumulation; (**B**) Macrovesicular steatosis reduction after simultaneous supplementation with GSPE; (**C**) Hematoxilin-eosin staining of representative histological sections of liver from STD, CAF, CAF PRE, and CAF MONTHLY rats. Scale bar = 10 μm in all pictures. STD: lean rats fed a standard diet; CAF: rats fed a cafeteria diet; CAF PRE: rats receiving preventive treatment of GSPE during 10 days before cafeteria diet intervention; CAF MONTHLY: rats receiving GSPE treatment during 5 days once per month simultaneously fed with cafeteria diet. Levels of triglycerides are represented as boxplots showing the median and the IQR. Means are represented as +. Macrovesicular steatosis score values are represented as means ± SEM. * *p*-value < 0.05 compared to STD rats. # *p*-value < 0.05 compared to CAF rats. Trends: ^$^ 0.05 < *p*-value < 0.1 compared to CAF rats.

An important characteristic of the metabolic syndrome associated with obesity is ectopic fat accumulation in the liver, which triggers hepatic steatosis. In our experiment, rats in the CAF group showed greater TAG accumulation in the liver than rats in the STD group (Figure 6A). Although preventive treatment with GSPE had no effect on diminishing TAG accumulation, the CAF MONTHLY group tended to reduce TAG levels compared to the CAF group (Figure 6A). To corroborate these data, we analysed fat accumulation to evaluate the presence of hepatic steatosis (Figure 6B). However, only macrovesicular steatosis was observed. As expected, the CAF group had a greater accumulation of lipid droplets in hepatocytes than the STD group (Figure 6C). Interestingly, although GSPE preventive treatment had no strong effect on reducing macrovesicular steatosis, when administered simultaneously, GSPE reduced diet-induced fat accumulation in the liver (Figure 6B,C).

### 3.6. Effect of GSPE on Systemic Inflammation in Aged Rats Fed an Obesogenic Diet

**Figure 7:**
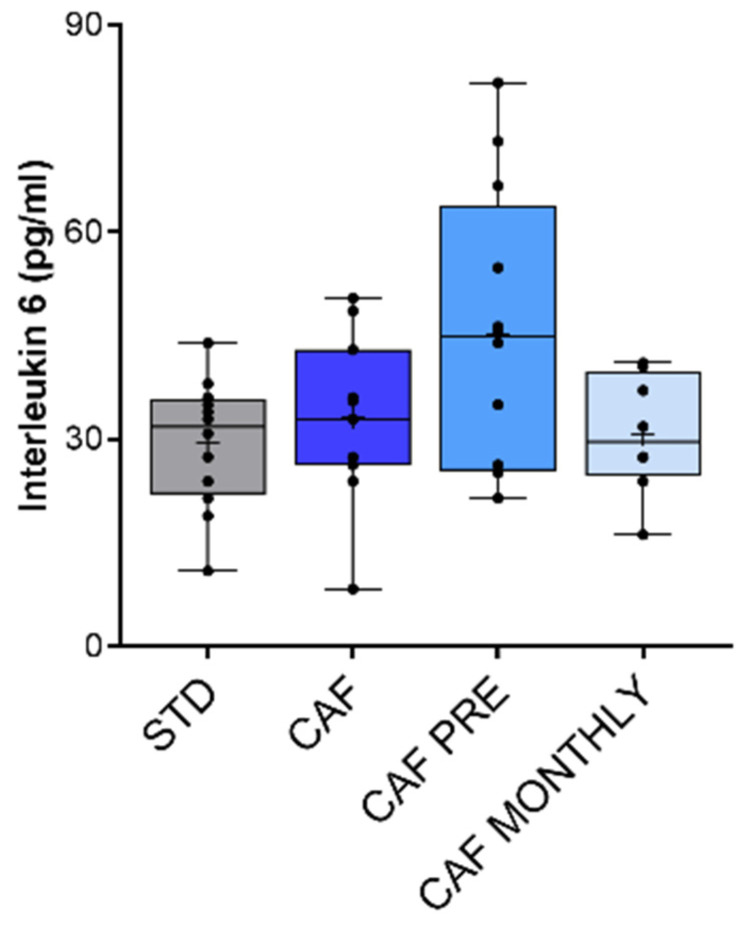
Interleukin-6 levels as biomarker of systemic inflammation. STD: lean rats fed a standard diet; CAF: rats fed a cafeteria diet; CAF PRE: rats receiving preventive treatment of GSPE during 10 days before cafeteria diet intervention; CAF MONTHLY: rats receiving GSPE treatment during 5 days once per month simultaneously fed with cafeteria diet. Values are represented as boxplots showing the median and the IQR. Means are represented as +.

Finally, we also measured TNF-α in plasma as a biomarker of systemic inflammation. TNF-α levels were undetectable in all samples analysed (data not shown). To further evaluate systemic inflammation in this model, we measured IL-6 levels in plasma. In agreement with TNF-α results, IL-6 levels were generally very low in aged rats. The CAF diet did not induce systemic inflammation in comparison with STD rats, and GSPE supplementation did not change inflammatory status (Figure 7).

## 4. Discussion

The prevalence of obesity is increasing in parallel with the worldwide growth in the aged population. This scenario makes it absolutely necessary to find new strategies aimed at mitigating the harmful effects of ultra-processed foods and improving our quality of life. Grape-seed proanthocyanidins may be good candidates for use as bioactive compounds against obesity, ageing, and related dysfunctions. Several studies have shown that GSPE has anti-inflammatory and anti-tumoral properties [29] and plays an important role in reducing hyperlipidemia [12,13]. Previous results from our group demonstrated that a dietary dose of 25 mg GSPE/kg of body weight was effective in improving obesity-associated intestinal damage and decreasing intestinal inflammation when administered as a corrective treatment in rats [30]. However, this dietary dose was not effective in decreasing body weight. In contrast, our group recently demonstrated that a dose of 500 mg/kg BW of GSPE is effective in reducing food intake in both lean and obese young rats [31] as well as in aged, chow-fed rats [15,32] and, therefore, helps to reduce body weight.

Additionally, from a functional food perspective, the most appropriate way for a compound to be effective as an anti-obesity agent is to prevent and/or approach the problem at the initial stages. As stated before, we have previously demonstrated the effectiveness of these preventive treatments of GSPE in young rats, and, now, the same hypothesis might be applied for the elderly. As far as we know, this study has, for the first time, assessed the effects of GSPE on most metabolic risk factors associated with obesity in aged rats. Our results support the hypothesis that grape-seed proanthocyanidins are effective in preventing certain metabolic syndrome features induced by an obesogenic challenge during ageing.

The animals in this study received an unhealthy, highly palatable, energy-dense human cafeteria diet. We have shown that the obesogenic diet administered to aged rats induced obesity and disturbed glucidic and lipidic profiles. More interestingly, a dose of 500 mg/kg BW of GSPE can prevent certain metabolic disruptions in aged rats, as has previously been demonstrated in young rats [15,31,33].

In our experiment, GSPE-treated rats in both the CAF PRE and CAF MONTHLY groups showed a decrease in body weight gain compared to rats in the CAF group. These results were previously observed by our group in a 17-week-experiment with young, female rats subjected to the same feeding conditions, GSPE dose, and administration method [31]. Although the durations of the two experiments are not comparable, aged rats fed the cafeteria diet had a higher body weight gain in a shorter period than young rats fed the same diet, indicating that aged rats are more susceptible to obesity. Various animal studies have also demonstrated that ageing is associated with higher body weight gain and fat mass accumulation. In agreement with our results, 16-month-old male rats showed greater body weight and adiposity than their young counterparts both under a normal chow diet and a high-fat diet before and after surgery or dietary interventions [34]. Interestingly, our results show that simultaneous GSPE treatment has the same efficacy in reducing body weight regardless of age. On the other hand, when administered as a preventive treatment, GSPE was not as effective in aged rats as it was in young rats. Despite the fact that GSPE-specific mechanisms of action to reduce adiposity and body weight gain have not been fully elucidated, out results in the present study suggest that effects of GSPE could be partially mediated by the decrease in food intake. Moreover, literature suggests that GSPE could induce white adipose tissue browning probably by acting as a scavenger receptor of free radicals, thus leading to a reduction of body weight [20].

GSPE is also widely reported to be a lipolytic agent [13,35,36]. For example, according to results obtained previously in young, female rats [31,37], in this work, GSPE reduced fat accumulation in the mesenteric adipose tissue of obese, aged rats when administered together with the cafeteria diet. However, no consensus could be established on the effect of GSPE on fat accumulation when administered preventively, which suggests that age may affect the long-term GSPE effect seen previously in young rats [31]. Dietary polyphenols, such as resveratrol and quercetin, are believed to promote longevity and life expectancy [38], while scientific evidence suggests they can also modulate epigenetic patterns [39,40]. Our group has also shown that the long-lasting effects of GSPE in young rats are mediated, at least partially, by epigenetic modifications in the intestine [41]. In the present study, the loss of long-lasting GSPE effects in aged animals may be due to the deep, epigenetic modifications that are linked to the ageing process [40]. Our results therefore suggest that aged animals are more prone to obesity and difficulty in losing weight. This may be due to age-related changes in body fat distribution and functional impairments [4,42] for which a short preventive GSPE treatment is insufficient to compensate for the homeostasis disruption associated with senescence.

In previous studies of young, female rats [37,43], all GSPE treatments ameliorated dyslipidaemia and the insulin-resistant state of obese rats. Other studies have also shown that GSPE protects against disorders in mice induced by a high-fat diet [44]. Contrary to our results, a dietary dose of GSPE (25 mg/kg BW) managed to reduce the plasma levels of triglycerides, glucose, and insulin in cafeteria-diet-fed male rats [45], while puerarin [46], resveratrol [47], and procyanidin B2 [17] ameliorated hyperglycaemia and hyperinsulinemia caused by an obesogenic diet. However, none of the above studies applied the double challenge of ageing and obesity, two factors that contribute to the dyslipemic and insulin-resistant phenotype. Indeed, our results may indicate that the cafeteria diet has a stronger effect on aged animals than on young ones, since aged animals have a marked increase in glucose and insulin levels in plasma probably due to the decline in adaptive homeostasis associated with age. The noticeable effect of the cafeteria diet on aged animals may therefore counteract the positive effects of GSPE on re-establishing homeostasis.

Together with inflammation and oxidative stress, hepatic steatosis is the basis for the pathophysiology of NALFD. Our results indicate that GSPE is effective in protecting against ectopic fat accumulation in the liver when administered simultaneously with the cafeteria diet. According to the literature, other bioactive compounds, such as procyanidin B2 [17,48], polyphenol-rich extract from cranberries [49], and myricetin [50], are good candidates for reducing fat accumulation in the liver. Specifically, resveratrol is one of the most promising polyphenols for improving lipid droplet accumulation in the liver in both young and aged male mice [51]. Several mechanisms, such as binding to bile acids [49,52], regulating the lysosomal pathway and redox state [53], activating free fatty acid β-oxidation, and modulating factors involved in lipid metabolism [54], are reported to be responsible for improving hepatic steatosis by dietary polyphenols. Some other authors pointed out that GSPE could influence expression of genes involved in different signaling pathways, such as glycolysis, insulin, or inflammatory pathways, via modulating microRNA expression in vitro [55]. Moreover, hepatocyte senescence increases with age, thus contributing to hepatic fat accumulation and steatosis [56]. Although the mechanisms behind this effect have not been explored in this article, modulation of the lipid metabolism and/or cellular senescence in the liver cannot be ruled out.

Contrary to previous results from studies that compared obese, young rats with lean rats [31,37], the cafeteria diet had no effect on increasing inflammatory response through an increase in the production of pro-inflammatory cytokines, especially TNF-α and IL-6. Interestingly, ageing had no effect on the serum levels of TNF-α or IL-6 in obese rats. These cytokines are usually considered biomarkers of ageing in humans [57], especially IL-6, IL-8, IL-1β, and TNF-α, since they are strong predictors of age-related morbidity and mortality [58,59]. Although relatively little is known about the identification of inflammatory biomarkers in rodent models of ageing, in agreement with our results, Gordon et al. [60] found that only 5 out of 58 pro-inflammatory-related molecules were modified in 24-month-old rats compared to 4-month-old rats. IL-6 and TNF-α were unchanged, and only C-reactive protein was increased.

Although most flavonoids and other bioactive compounds are reported to have an anti-inflammatory effect, in our model, we saw no such effect with GSPE. These results might be related with the decline in immunological response associated with immunosenescence during ageing [61]. Immunosenescence is defined as a progressive and overall diminution of immune functions that affect all cells and organs, where defects in the processing and presentation of antigens by the cells of the innate immune system contribute to diminished activation and stimulation of cells in the adaptive immune system [57]. Furthermore, the progressive involution of the thymus leads to a disturbed balance and function of naïve, memory, and effector T cells, thus promoting a latent pro-inflammatory status in the elderly, called inflammageing [58]. This progressive proinflammatory state in the elderly has been described as elevated plasma concentrations of IL-6, IL-1β, and TNF-α, among other cytokines [62]. The inflammageing process seems to call in question the functional defects observed in innate immune cells. However, it is believed that chronic, subclinical inflammation is caused by the chronic antigenic stress that impinges throughout life upon innate immunity and/or by the partial inability of the aged immune system to eliminate certain pathogens. This could lead to chronic, yet inefficient, innate immune responses and has potential implications for the onset of inflammatory diseases [63,64].

However, as previously stated, we have not found this low-grade, pro-inflammatory state in our model of obese/aged rats nor as an effect of GSPE treatment. A hypothesis to explain these results might be the fact that the rats used in our experiment were not under any substantial chronic stimulation of the innate immune system, as they were grown in the animal house during their whole life. Without this chronic stimulus, it is possible that the pro-inflammatory state in the elderly was not developed. From this perspective, our model is not ideal to study immunosenescence. In any case, better understanding of age-associated changes in the immune system should enable the development of more effective strategies to promote a healthy ageing.

In summary, GSPE treatment is not as effective in old age as it is in youth. This is probably due to the great metabolic disruption associated with the ageing process and the inability to respond quickly to homeostatic fluctuations. With this in mind, GSPE should be administered before the normal functioning of the organism declines over time.

## 5. Conclusions

In conclusion, administering GSPE at a dose of 500 mg/kg BW prevents the development of certain unhealthy states related to obesity and ageing. Simultaneous treatment with GSPE is effective in reducing body weight gain, adiposity, and liver steatosis caused by cafeteria-diet consumption.

Further studies are needed to determine the potential memory effect of GSPE during ageing and under an obesogenic challenge. The final goal is to translate these approaches to human nutrition by developing new strategies based on bioactive agents to improve the quality of life of elderly people. However, the time of administration and optimal dose must be ascertained.

## Figures and Tables

**Table 1:** Composition of the cafeteria diet offered.

Component Offered	kJ/g	% Carbohydrate (g)	% Protein (g)	% Lipid (g)	% Fiber (g)
Bacon	14.43	1.0	14.9	31.7	0.0
Sausages	8.36	8.0	14.0	18.0	0.0
Paté	6.57	0.7	8.5	11.0	0.0
Biscuits	18.4	22.0	6.6	10.3	2.0
Muffins	18.8	30.0	4.1	23.1	1.7
Carrot	1.66	0.7	0.1	0.0	2.6
Milk	2.74	4.7	3.1	3.8	0.0
Sugar	16.73	100.0	0.0	0.0	0.0
STD chow diet	12.13	48.0	14.3	4.0	4.1

STD, standard chow provided to the control group.

**Table 2:** Morphometric variables of the groups studied.

Variable	STD	CAF	CAF PRE	CAF MONTHLY
*n*	13	12	12	8
Morphometric Measurements				
Initial body weight (g)	364.0 ± 14.2	366.4 ± 11.8	355.7 ± 9.6	352.36 ± 2.5
Final body weight (g)	367.4 ± 15.0	516.0 ± 20.1 *	470.2 ± 17.2	472.3 ± 18.9
mWAT (g)	13.1 ± 1.2	28.1 ± 2.0 *	21.0 ± 1.5 ^#^	18.8 ± 1.4 ^#^
oWAT (g)	16.6 ± 1.5	31.7 ± 1.2 *	31.8 ± 2.3	27.5 ± 2.3
rWAT (g)	11.1 ± 1.1	22.2 ± 1.2 *	20.3 ± 1.2	19.3 ± 1.2
Total visceral WAT (g)	39.5 ± 3.4	80.7 ± 4.9 *	72.1 ± 4.2	65.8 ± 2.4 ^$^
BAT (g)	0.7 ± 0.1	1.3 ± 0.1 *	1.1 ± 0.1	1.5 ± 0.4
% visceral adiposity	11.3 ± 0.6	16.3 ± 0.5 *	15.4 ± 0.6	14.0 ± 0.5 ^#^
Liver (g)	8.7 ± 0.4	12.2 ± 0.8 *	11.2 ± 0.6	10.9 ± 0.3
Spleen (g)	0.8 ± 0.0	1.0 ± 0.0 *	0.9 ± 0.1	0.9 ± 0.0
Kidney (g)	1.0 ± 0.0	1.2 ± 0.1 *	1.2 ± 0.0	1.1 ± 0.0

STD: lean rats fed a standard chow diet; CAF: rats fed a cafeteria diet; CAF PRE: rats receiving a GSPE preventive treatment 10 days before the cafeteria intervention; CAF MONTHLY: rats receiving a GSPE treatment during 5 days synchronized with the cafeteria diet; mWAT, mesenteric white adipose tissue; oWAT, periovaric white adipose tissue; rWAT, retroperitoneal white adipose tissue; WAT, white adipose tissue; BAT, brown adipose tissue. Values are means ± SEM. * *p* < 0.05 compared to STD group. ^#^ *p* < 0.05 compared to CAF group; Trends: ^$^ 0.05 < *p*-value < 0.1 compared to CAF rats.
